# Involving children in disaster risk reduction: the importance of participation

**DOI:** 10.1080/20008198.2018.1425577

**Published:** 2018-02-05

**Authors:** Betty Pfefferbaum, Rose L. Pfefferbaum, Richard L. Van Horn

**Affiliations:** ^a^ Department of Psychiatry and Behavioral Sciences, College of Medicine, University of Oklahoma Health Sciences Center, Oklahoma City, OK, USA; ^b^ Liberal Arts Department, Phoenix Community College, Phoenix, AZ, USA; ^c^ Management Information Systems, Aviation and Aerospace Studies, University of Oklahoma, Norman, OK, USA

**Keywords:** Child development, children, children’s rights, disaster, disaster management, disaster preparedness, disaster risk reduction, participation, youth, desarrollo infantil, niños, derechos de los niños, desastres, gestión de desastres, preparación para desastres, reducción del riesgo de desastres, participación, juventud, 儿童发展, 儿童, 儿童权力, 灾害, 灾害管理, 灾害准备, 灾害风险减少, 参与, 青年, • Children are resources to be cultivated and mobilized for disaster preparedness, response, recovery, and resilience., • Children’s participation yields numerous potential benefits for children, including enhanced personal development and skills, self-efficacy, and interpersonal relationships., • Children’s participation yields numerous potential benefits for communities through improved social connections and networks and disaster preparedness., • Attention is needed to identify approaches to appropriately involve children in disaster risk reduction activities, to promote these efforts, and to evaluate these approaches.

## Abstract

**Background**: Millions of children are affected by disasters every year. Children need not be passive victims, however, but instead may contribute to disaster risk reduction activities.

**Objective**: This paper provides a theoretical foundation for children’s involvement in disaster risk reduction activities.

**Method**: The paper reviews and analyses the literature on children’s participation, on their developmental capacity to participate, and on disaster risk reduction activities involving children.

**Results**: Participation yields numerous potential benefits for children, including enhanced personal development and skills, self-efficacy, and interpersonal relationships, and for communities through improved social connections and networks and disaster preparedness.

**Conclusions**: Children are resources to be cultivated and mobilized for disaster preparedness, response, recovery, and resilience. Attention is needed to identify approaches to appropriately enlist, engage, and involve children in disaster risk reduction activities; to promote these efforts; and to evaluate these approaches.

## Abstract (es)

**Planteamiento**: Millones de niños se ven afectados por desastres cada año. Sin embargo, los niños no necesitan ser víctimas pasivas, sino que pueden contribuir a las actividades de reducción del riesgo de desastres.

**Objetivo**: Este documento proporciona una base teórica para la participación de los niños en actividades de reducción del riesgo de desastres.

**Método**: El documento revisa y analiza la literatura sobre la participación de los niños, acerca de su capacidad de desarrollo para participar y de las actividades de reducción del riesgo de desastres que involucran a los niños.

**Resultados**: la participación ofrece numerosos beneficios potenciales tanto para los niños, que incluyen una mejoría de su desarrollo personal y habilidades, de su autoeficacia y de sus relaciones interpersonales, como para las comunidades a través de mejores conexiones y redes sociales, y una mejor preparación para los desastres.

**Conclusiones**: Los niños son recursos que se pueden cultivar y movilizar para la preparación, la respuesta, la recuperación y la resiliencia ante desastres. Se necesita atención para identificar enfoques para reclutar, involucrar e implicar apropiadamente a los niños en actividades de reducción del riesgo de desastres; para promover estos esfuerzos; y para evaluar estos enfoques.

## Abstract (zh)

**背景：**每年有数以百万的儿童被灾害影响。儿童不应该成为被动受害者，反而应该参与到灾害风险减少的行动中。

**目标：**本文提供了一个让儿童参与到灾害风险减少行动中的理论基础。

**方法：**本文综述和分析了关于儿童参与、儿童的发展性参与能力，和涉及儿童的灾害风险减少行动三个方面的文献。

**结果：**参与本身给儿童带来了大量可能的好处，包括增加个人发展和技能、自我效能感和人际关系。同时也给社区带来了增进社会联结、社交网络以及灾害准备这些方面的好处。

**结论：**对儿童进行培养和动员，他们会成为灾害准备、反应、恢复和抗逆性的源头。需要有意识地寻找方法招募、吸引儿童并鼓励其参与到灾害风险减少行动中，促进这些努力并对这些方法进行评估。

## 1. Introduction

Millions of children throughout the world are affected by disasters each year (Penrose & Takaki, 2006). Children, especially those living in poverty or in marginal and underdeveloped environments, assume a disproportionate share of the burden created by disasters, both in the near and long term (Back, Cameron, & Tanner, 2009). In 2011, declaring children to be ‘the group most affected by disasters each year’, the United Nations International Strategy for Disaster Reduction (UNISDR, 2011, p. 1) advocated their active participation in disaster risk reduction activities. Disaster risk reduction represents organized approaches to examine and address factors associated with disaster risk and exposure, with human and material vulnerabilities, and with adverse disaster effects (UNISDR, 2009). Rather than simply passive victims of disasters (Anderson, 2005; Mitchell, Haynes, Hall, Choong, & Oven, 2008; Peek, 2008), children can contribute to disaster mitigation, preparedness, and response (Anderson, 2005). Thus, children are resources to be nurtured and mobilized in support of disaster preparedness and resilience for the present and future (US Department of Health and Human Services [USDHHS], 2017).

The literature on child participation speaks to the role of children in activities, programmes, institutions, and/or processes that engage them in issues that concern them and in decision making about those issues (O’Donoghue, Kirshner, & McLaughlin, 2002). Despite substantial literature across various disciplines that supports children’s participation, including reports of children’s involvement in activities related to climate change and disasters (e.g. Back et al., 2009; Federal Emergency Management Agency [FEMA], 2015; Peek, 2008), child involvement in disaster risk reduction activities has not been well covered in the mental health literature. This paper explores the literature on child participation in general to establish a theoretical foundation for children’s involvement in the disaster context. The paper presents support for involving children, considers children’s developmental capacity to participate and the involvement and importance of adults in youth participation, and reviews disaster applications and the potential benefits of youth participation for both children and their communities.

## 2. Support for involving children

Support for children’s participation is found in social (e.g. consumerism) and health-care (e.g. shared decision making) movements; in the recognition of children’s social contributions; in evidence that children, including very young children, can contribute actively to their social environments through observation, inquiry, evaluation, and decision making; and in findings that their involvement can advance social and democratic processes, inform and improve decision making, reinforce children’s connections and commitment to their communities, and increase awareness of their needs and desires (Sinclair, 2004). The children’s rights agenda, exemplified in the Convention on the Rights of the Child, a global pledge to advance the protection, welfare, and rights of children regardless of gender, race, religion, or ability, also champions child participation (United Nations General Assembly, 1989). Adopted by the United Nations General Assembly in 1989, the seminal Convention on the Rights of the Child requires that children be included as collaborators in activities related to their well-being (Chawla & Driskell, 2006). While the extent to which the Convention on the Rights of the Child has been effectively implemented is unclear, it is widely believed that its promulgation has led to the recognition that children are valuable resources who can make meaningful contributions in many areas (Chawla & Driskell, 2006).

## 3. Developmental considerations in children’s participation

Children’s participation is influenced by their evolving cognitive development and socio-cultural expectations and opportunities (Hart, 1997; Wong, Zimmerman, & Parker, 2010). Pfefferbaum, Van Horn, and Pfefferbaum (in press) presented the theoretical rationale and offered an approach for involving adolescents in enhancing disaster-related community resilience which has application for other disaster risk reduction activities as well. Reviewing adolescent development, they elucidated youth cognitive and social readiness for participation in these activities. To the extent that activities are developmentally appropriate and adult involvement is available to support their efforts, younger children can participate in disaster management activities as well. School-age children are enthusiastic and industrious, and they have the potential to share and work with others. Participation in organized activities can promote children’s industry in the face of potential failure and ward against the development of a sense of inferiority (Hart, 1997).

Among other things, children develop through involvement with others as they discover who they are and who they want to become (Wong et al., 2010). Children are competent and motivated to work together to plan, organize, and manage complicated projects when they feel some ownership in them. In turn, their involvement can further motivate them and reinforce skill development. Moreover, opportunities for interaction with adults who provide guidance and support can facilitate the maturation of children’s competence. With some limitations, children of early school age are able to work with adults. In some settings, children can take the lead in community participation and can even stimulate adult involvement (Hart, 1992). Children are able to participate with multiple generations as well, with older youth often serving as intermediaries (Wong et al., 2010).

Participation depends in part on the child’s ability to take the perspective of others (Hart, 1997). Hart (1997) observed that by the age of three years, children have limited ability in this regard with continued development in this capacity through adolescence. As they mature, children increasingly recognize that people have their own personal views, reflect on their own interactions and appreciate that others can do so also, and realize that people can have many and mixed feelings. Adolescents appreciate the possibility that individuals may share perspectives and that people use shared perspectives to foster communication and understanding (Hart, 1997).

## 4. The role of adults

Adult opposition to children’s involvement stems from concern that youth are not able to participate or interested in doing so (Matthews, 2003) and that the time and effort involved in implementing and supporting child participation may decrease the time and effort needed for other important endeavours (Lundy, 2007) as well as the view that parents are responsible for making decisions about their children and for informing and protecting their children (Mitchell et al., 2008). These parental obligations do not preclude children’s participation, however, and children’s participation does not obviate parental or adult responsibility. In fact, adults have both a role and a responsibility in guiding and directing child participation (Lundy, 2007) and in offering social support and supervision (Wong et al., 2010). Adults serve as important role models and as resources to encourage awareness, critical dialogue, and skill enhancement when youth share their views regarding issues about which they are knowledgeable (Wong et al., 2010). Adults are needed to help establish networks that provide influence, commitment, and resources to facilitate the acceptance, application, and implementation of children’s ideas and recommendations (Chawla & Driskell, 2006). Because of their superior skills, expertise, and connections, adult leadership may improve activities, programmes, and outcomes (Wong et al., 2010).

## 5. Disaster applications

Increased awareness and knowledge of local threats and active involvement in disaster risk reduction among all community members – including children – are needed to create a ‘culture of risk reduction’ (Morris & Edwards, 2008, p. 389). While children’s vulnerability in the face of natural disasters is well established, their involvement in disaster management has received relatively little attention even though ignoring their possible role in disaster risk reduction can endanger them in the event of a disaster and overlooks a potential resource for the communities where they live (Mitchell et al., 2008). Informed and engaged children may be better able than those who lack information and are unengaged to protect themselves and others (Peek, 2008).

Parents and other adults may not appreciate children’s concerns (Chawla & Driskell, 2006) in the context of disasters and they tend to underestimate children’s disaster reactions (Peek, 2008). Thus, children should be queried about their understanding of events, their reactions, and their concerns and priorities (Peek, 2008). In addition, children can provide useful original and constructive information about their communities and about how to assist their families and communities (FEMA, 2015; Peek, 2008). Disaster preparedness should include efforts to educate children about disasters and available resources (Peek, 2008) and to provide developmentally appropriate information about potential hazards and risks, how children can prepare for events, what to do in the face of an event, and what they might need from adults (Mitchell et al., 2008).

Teaching children about natural hazards promotes their involvement in preparedness, response, and recovery (Morris & Edwards, 2008). Because youth are commonly creative, idealistic, and passionate, they can be effective advocates for preparedness by helping disseminate information (USDHHS, 2017) to educate and prepare family and friends (FEMA, 2015) thus enhancing resilience and recovery in themselves, their families, and their communities (Peek, 2008). Specifically, children can participate in a variety of preparedness activities including hazard and threat identification, drills, evacuation planning, home adjustments, search and rescue training, and risk communication; in response efforts such as risk communication, evacuation, and search and rescue; and in recovery efforts such as helping to care for others, gathering and distributing resources, peer counselling, planning and reconstruction projects, and assisting with child care and household responsibilities (Peek, 2008). Meaningful participation is best developed through practice rather than taught in the abstract (Hart, 1992) and may be most effective when children participate in experiential activities (Peek, 2008). Schools and other community-sponsored efforts can offer both formal and informal disaster preparedness, response, and recovery activities (Peek, 2008). See Table 1 for sample activities for children’s participation in preparedness, response, and recovery.

**Table 1.** Sample youth activities across phases of disaster management.

Examples by selected categories^a^	Disaster management phases		
	Preparedness	Response	Recovery
**Education and training**			
Learn about disasters and risk reduction	√	√	√
Become familiar with the disaster system of care	√	√	√
Train to serve on neighbourhood disaster response team	√	√	
Participate in local disaster drills	√		
Create a disaster charades game	√	√	√
Develop relaxation skills	√	√	√
Build family, school, and community awareness	√	√	√
Educate family, peers, and others	√	√	√
**Assessment**			
Help create and conduct surveys of school, neighbourhood, and community	√	√	√
Prepare neighbourhood infrastructure maps	√		
Identify likely threats in the local area	√		√
Canvas neighbourhood to ascertain damages		√	√
Explore family, peer, and community values	√		√
Enter information into databases	√	√	√
**Planning**			
Participate in family, school, neighbourhood, and community planning sessions	√	√	√
Plan for pets	√	√	√
Engage senior citizens and other special groups in planning	√	√	√
**Information gathering and dissemination**			
Conduct web searches	√	√	√
Identify helpful mobile apps	√	√	√
Create identification cards with key contact information	√		√
Prepare resource directories of locally available services	√	√	√
Visit stakeholder organizations to discuss potential roles in disaster management	√	√	√
Identify age-appropriate volunteer opportunities	√	√	√
Identify and share evacuation routes and procedures	√		
Prepare mailings	√	√	√
Make posters addressing disaster related issues	√	√	√
**Community narrative**			
Develop a historical timeline of prior community disasters	√		√
Interview community members regarding their experiences with former and current local disasters	√	√	√
Create murals depicting the local community	√	√	√
**Communication**			
Add emergency contact information to telephones	√	√	√
Create family communication plans	√		√
Craft and deliver safety messages	√	√	√
Promote disaster management in person and via social and print media	√	√	√
Write and act in skits related to disasters, disaster management activities, or the community	√	√	√
**Service delivery**			
Help staff volunteer centres and operations		√	√
Assist at shelters		√	√
Distribute water, food, and/or supplies		√	√
Identify organizations that family or peers can access for assistance	√	√	√
Assist with younger children		√	√
Engage in clean-up campaigns		√	√
Provide peer counselling		√	√
**Programme evaluation**			
Assist in programme monitoring	√	√	√
Disseminate and collect programme evaluation forms	√	√	√
Explain the importance of evaluation to family, peers, and community members	√	√	√
**Critical analysis**			
Provide a youth perspective via focus groups, community conversations, and interviews	√	√	√
Help interpret quantitative and qualitative data within the context of the local environment	√	√	√
Conduct stakeholder analysis	√	√	√
Contribute to ecological mapping	√		√
**Other**			
Establish and serve on youth councils	√	√	√
Discuss and evaluate coping mechanisms used by peers in stressful situations	√	√	√
Identify supplies needed in an emergency kit and explain their purpose	√		√
Make disaster kits	√		√
Create collages depicting responders	√	√	√
Solicit funding and other support from local businesses and civic groups	√	√	√

^a^The assignment of general and specific examples to categories is illustrative rather than definitive since some examples may be relevant in multiple categories.

## 6. Benefits of child participation

A number of benefits – for both children and communities – accrue from children’s participation. As they try to find their role in society, youth need opportunities to participate in meaningful activities. In fact, children commonly express their motivation for participation as the desire to make a difference (Fleming, 2013). Engaging youth in collaborative activities and decision making exposes them to multiple perspectives, critical deliberation, analytic and problem-solving approaches, and strategic planning, and it supports cognitive and social development (Wong et al., 2010). Formal and informal disaster efforts provide opportunities to increase children’s awareness and knowledge and to enhance their personal confidence, self-esteem (Chawla & Heft, 2002), sense of social worth (Matthews, 2003), connectedness (Chawla & Heft, 2002), and personal- and collective-efficacy (Chawla & Heft, 2002; Jennings, Parra-Medina, Messias, & McLoughlin, 2006). Thus, participation in disaster risk reduction activities supports empowerment in children which in turn should enhance their resilience. Moreover, participation may promote children’s development more generally by teaching them to accept and adapt to change (USDHHS, 2017).

Participation in community efforts creates opportunities for adult–child interactions to build relationships (Matthews, 2003), recognize each other’s strengths and assets, and appreciate the views and needs of others (Chawla & Heft, 2002; Jennings et al., 2006) and the value of partnership and collaboration (Jennings et al., 2006). Engaging children also has direct benefit for the community through, for example, enhanced social connection and networks and an informed and involved citizenry (Chawla & Heft, 2002; Matthews, 2003), which may lead to a better prepared populace. Unfortunately, little scientific evidence has emerged to support these potential benefits of children’s participation (Chawla & Heft, 2002) or of children’s involvement in disaster risk reduction activities (Back et al., 2009). Successful outcomes for both children and their communities depend on the choice of activities, the creation of and advancement toward programme goals, and the relationships established with informed and supportive adults (USDHHS, 2017).

## 7. Conclusions

Disasters affect millions of children worldwide each year. While children’s vulnerability and needs are widely recognized, they are not passive victims. Since children are able to contribute to disaster risk reduction activities, they represent valuable resources to nurture and mobilize for disaster preparedness, response, recovery, and resilience at the individual, family, and community level. Children’s participation in disaster risk reduction is supported by the recognition of their social competence, by the children’s rights agenda, and by a literature advancing their participation in a variety of community endeavours. Children’s participation in disaster risk reduction yields potential benefits for children through enhanced personal development and skills, self-efficacy, and interpersonal relationships and for communities through improved social connections and networks and disaster preparedness. Unfortunately, empirical evidence on youth involvement in disaster risk reduction activities is lacking. Important next steps include identifying, applying, and evaluating approaches and implementation models that appropriately enlist, engage, and involve children in disaster risk reduction activities. Also needed is an understanding of the barriers and challenges to children’s participation and of potential harms that stem from their involvement.

## References

[CIT0001] Anderson W. A. (2005). Bringing children into focus on the social science disaster research agenda. *International Journal of Mass Emergencies and Disasters*, 23(3), 159–6.

[CIT0002] Back E., Cameron C., & Tanner T. (2009). *Children and disaster risk reduction: Taking stock and moving forward*. Brighton: Institute of Development Studies Retrieved from http://www.ids.ac.uk/publication/children-and-disaster-risk-reduction-taking-stock-and-moving-forward

[CIT0003] Chawla L., & Driskell D. (2006). The *Growing Up in Cities* project: Global perspectives on children and youth as catalysts for community change. *Journal of Community Practice*, 14(1–2), 183–200.

[CIT0004] Chawla L., & Heft H. (2002). Children’s competence and the ecology of communities: A functional approach to the evaluation of participation. *Journal of Environmental Psychology*, 22(1–2), 201–216.

[CIT0005] Federal Emergency Management Agency (2015). *National strategy for youth preparedness education. Empowering, educating and building resilience*. Washington, DC: US Department of Homeland Security Retrieved from https://www.fema.gov/media-library/assets/documents/96107

[CIT0006] Fleming J. (2013). Young people’s participation – Where next? *Children and Society*, 27, 484–495.

[CIT0007] Hart R. A. (1992). *Children’s participation. From tokenism to citizenship* (Innocenti Essays No. 4). Florence: UNICEF International Child Development Centre Retrieved from https://www.unicef-irc.org/publications/pdf/childrens_participation.pdf

[CIT0008] Hart R. A. (1997). Children’s developing capacity to participate In Hart R. A. (Ed.), *Children’s participation: The theory and practice of involving young citizens in community development and environmental care* (pp. 27–39). New York, NY: UNICEF/Earthscan Publications Ltd., Taylor and Francis.

[CIT0009] Jennings L. B., Parra-Medina D. M., Messias D. K. H., & McLoughlin K. (2006). Toward a critical social theory of youth empowerment. *Journal of Community Practice*, 14(1–2), 31–55.

[CIT0010] Lundy L. (2007). ‘Voice’ is not enough: Conceptualising Article 12 of the United Nations convention on the rights of the child. *British Educational Research Journal*, 33(6), 927–942.

[CIT0011] Matthews H. (2003). Children and regeneration: Setting an agenda for community participation and integration. *Children & Society*, 17(4), 264–276.

[CIT0012] Mitchell T., Haynes K., Hall N., Choong W., & Oven K. (2008). The roles of children and youth in communicating disaster risk. *Children, Youth and Environments*, 18(1), 254–279. Retrieved from http://www.jstor.org/stable/10.7721/chilyoutenvi.18.issue-1

[CIT0013] Morris K. N., & Edwards M. T. (2008). Disaster risk reduction and vulnerable populations in Jamaica: Protecting children within the comprehensive disaster management framework. *Children, Youth and Environments*, 18(1), 389–407. Retrieved from http://www.jstor.org/stable/10.7721/chilyoutenvi.18.1.0389

[CIT0014] O’Donoghue J. L., Kirshner B., & McLaughlin M. (2002). Introduction: Moving youth participation forward In *New directions for youth development: Theory, practice and research, No. 96* (pp. 15–26). San Francisco, CA: Jossey-Bass (Wiley Periodicals, Inc.). doi:10.1002/yd.24 12630271

[CIT0015] Peek L. (2008). Children and disasters: Understanding vulnerability, developing capacities, and promoting resilience – An introduction. *Children, Youth and Environments*, 18(1), 1–29. Retrieved from http://www.jstor.org/stable/10.7721/chilyoutenvi.18.issue-1

[CIT0016] Penrose A., & Takaki M. (2006). Children’s rights in emergencies and disasters. *Lancet*, 367(9511), 698–699. 16503472 10.1016/S0140-6736(06)68272-X

[CIT0017] Pfefferbaum B., Van Horn R. L., & Pfefferbaum R. L. (in press). Involving adolescents in building community resilience for disasters. *Adolescent Psychiatry*.

[CIT0018] Sinclair R. (2004). Participation in practice: Making it meaningful, effective and sustainable. *Children & Society*, 18(2), 106–118.

[CIT0019] US Department of Health and Human Services, Office of the Assistant Secretary for Preparedness and Response (2017). *NPRSB-NACCD joint youth leadership report*. Washington, DC: Author Retrieved from https://www.phe.gov/Preparedness/legal/boards/nprsb/meetings/Documents/joint-youth-ldrshp-rpt.pdf

[CIT0020] United Nations General Assembly (1989, 11 20). Convention on the rights of the child. United Nations, Treaty Series (Vol. 1577). Retrieved from http://www.ohchr.org/EN/ProfessionalInterest/Pages/CRC.aspx

[CIT0021] United Nations International Strategy for Disaster Reduction (2009). *UNISDR terminology on disaster risk reduction*. Geneva: United Nations Retrieved from https://www.unisdr.org/we/inform/terminology

[CIT0022] United Nations International Strategy for Disaster Risk Reduction (2011, 10 13). UNISDR says the young are the largest group affected by disasters. Retrieved from https://www.unisdr.org/archive/22742

[CIT0023] Wong N. T., Zimmerman M. A., & Parker E. A. (2010). A typology of youth participation and empowerment for child and adolescent health promotion. *American Journal of Community Psychology*, 46(1–2), 100–114. 20549334 10.1007/s10464-010-9330-0

